# High Content Screening Using New U2OS Reporter Cell Models Identifies Harmol Hydrochloride as a Selective and Competitive Antagonist of the Androgen Receptor

**DOI:** 10.3390/cells9061469

**Published:** 2020-06-16

**Authors:** Hadjer Dellal, Abdelhay Boulahtouf, Elina Alaterre, Alice Cuenant, Marina Grimaldi, William Bourguet, Céline Gongora, Patrick Balaguer, Philippe Pourquier

**Affiliations:** 1 IRCM, Institut de Recherche en Cancérologie de Montpellier, INSERM U1194, F-34298 Montpellier, France; hadjer.dellal@inserm.fr (H.D.); abdel.boulahtouf@icm.unicancer.fr (A.B.); elina.alaterre@igh.cnrs.fr (E.A.); alice.cuenant@hotmail.fr (A.C.); marina.dilillo-grimaldi@inserm.fr (M.G.); celine.gongora@inserm.fr (C.G.); 2 Université de Montpellier, F-34298 Montpellier, France; william.bourguet@cbs.cnrs.fr; 3 Institut régional du Cancer de Montpellier, F-34298 Montpellier, France; 4 Centre de Biochimie Structurale, CNRS, INSERM, Université de Montpellier, F-34298 Montpellier, France

**Keywords:** prostate cancer, resistance to castration, androgen receptor, antagonists, harmol hydrochloride, high content screening

## Abstract

Prostate cancer is the most commonly diagnosed malignancy in men. Its growth mainly relies on the activity of the androgen receptor (AR), justifying the use of androgen deprivation therapy as a gold standard treatment for the metastatic disease. Inhibition of the androgen axis using second generation antagonists has improved patients’ survival, but is systematically confronted to resistance mechanisms, leading to a median survival that does not exceed 5 years. Counteracting this resistance has been the object of a large number of investigations, with a particular emphasis towards the identification of new AR inhibitors, whether they antagonize the receptor by a competitive or a non-competitive binding. To this end, many high content screens have been performed, to identify new non-steroidal AR antagonists, using a variety of approaches, but reported somewhat controversial results, depending on the approach and on the cell model that was used for screening. In our study, we used the U2OS osteosarcoma cells stably transfected with AR or ARv7 and a luciferase reporter as a previously validated model to screen the Prestwick Phytochemical library. The results of our screen identified ellipticine, harmol, and harmine hydrochloride as confirmed hits. Surprisingly, we could demonstrate that harmol hydrochloride, previously identified as a non-competitive inhibitor of AR or a weak inhibitor of androgen signaling, was actually a competitive antagonist of AR, which inhibits the growth of VCaP prostate cancer line, at concentrations for which it did not affect the growth of the AR negative DU145 and PC3 cells. Interestingly, we also report for the first time that harmol hydrochloride was selective for AR, as it could not alter the activity of other nuclear receptors, such as the glucocorticoid receptor (GR), the progesterone receptor (PR), or the mineralocorticoid receptor (MR). Additionally, we demonstrate that, conversely to enzalutamide, harmol hydrochloride did not show any agonistic activity towards the pregnane X receptor (PXR), a master regulator of drug metabolism. Together, our results shed light on the importance of the cellular context for the screening of new AR antagonists. They further indicate that some of the potential hits that were previously identified may have been overlooked.

## 1. Introduction

Prostate cancer is the most commonly diagnosed malignancy in men and a major health issue, as it represents the third highest cause of death by cancer in industrialized countries [1]. The proliferation of prostate tumor cells relies on the transcriptional activity of the androgen receptor (AR), which closely depends on the levels of its ligands testosterone or dihydrotestosterone (DHT). Upon the binding of androgens to its ligand binding domain (LBD), AR operates a conformational change in the cytoplasm, which releases protein chaperones, and induces a dimerization of the receptor. Activated AR is then translocated into the nucleus, where it binds to specific androgen response elements (ARE), via its DNA binding domain (DBD) in the promoter region of its target genes. This results in either activation or repression of their transcription, depending on whether AR interacts with co-activators or co-repressors, respectively [2]. 

Inhibition of the androgen axis by androgen deprivation therapy (ADT) is the predominant strategy for the treatment of metastatic prostate cancer. It can be achieved surgically (orchiectomy) or pharmacologically, using agonists or antagonists of GnRH to block the testicular synthesis of androgens. However, despite low levels of circulating testosterone, disease progression is invariably observed, leading to a castration-resistant state, in which tumor growth relies on the intracrine production of androgens. This justified the development of AR antagonists that prevent AR signaling by inhibiting the ligand binding to the receptor, or by inhibiting the translocation of AR to the nucleus, and its subsequent binding to ARE sequences on the DNA. Though a significant improvement of survival has been observed with the approval of first (bicalutamide, hydroxyflutamide) and second generation non-steroidal AR antagonists (enzalutamide, apalutamide), resistance to these agents is also observed. Several AR-dependent mechanisms have been described to explain this resistance (see [3,4,5,6,7,8] for recent reviews). They include the amplification and/or overexpression of the receptor [9]; point mutations that convert AR antagonists into agonists [10,11]; expression of splice variants lacking the LBD, such as ARv7 that is constitutively active [12]; and was involved in the clinical resistance to enzalutamide and abiraterone acetate [13]; the activation of AR by other ligands than androgens [14]; or the overexpression of coactivators of the receptor [2,15]. 

Counteracting this resistance has been the object of a large number of investigations, with a particular emphasis on working towards the identification of new AR inhibitors, whether they antagonize AR by a competitive binding to the LBD, or to specific domains of the receptor, including AF2, BF3, the nuclear localization signal, the N-terminal domain, or the DNA binding domain, or whether they inhibit AR in a non-competitive manner. Though there is no antagonist-bound AR crystal structure yet available, a number of recent studies were performed using molecular docking approaches or structure and ligand-based virtual screening [16,17,18,19]. Other studies relied on de novo synthesis, based on structure-based design or structure-activity relationship data [20,21,22,23], on chemical genomic approaches [24], or on more conventional cell-based high content screens of chemical libraries from various origins, in which AR inhibition was quantified using the transcription of the AR reporter genes [25,26,27,28,29], AR ligand-induced conformation change [26], or a reduction of AR nuclear localization [22] as readouts. While new non-steroidal derivatives with high inhibitory potential were identified, and are the subject of early clinical trials (reviewed in [30]), several hits identified by high content screening have been reported with somehow controversial results, depending both on the approach and on the cell model that was used for screening. Here, we have used osteosarcoma U2OS cell lines stably expressing the full-length wild-type AR or its ARv7 variant, in which it is possible to quantify the effect of drug candidates on the transcriptional activity of the receptor, using a luciferase-based reporter system. This model has been demonstrated to efficiently and specifically evaluate interferences of any chemical on nuclear receptors activity [27].

Using these models, we screened the Prestwick Natural Compounds Library, as many phytochemicals have been reported to modulate AR both at the expression and at the functional level [31]. We found that harmol hydrochloride, previously identified as a non-competitive inhibitor of AR [25,26] or a weak inhibitor of androgen signaling [24], is actually a competitive antagonist of AR that inhibits the growth of the VCaP prostate cancer line, at concentrations for which it did not affect the growth of AR negative DU145 and PC3 cells. Interestingly, we also report for the first time that harmol hydrochloride was selective for AR, as it could not alter the activity of other nuclear receptors such as the glucocorticoid receptor (GR), the progesterone receptor (PR), or the mineralocorticoid receptor (MR). Additionally, we demonstrate that, conversely to enzalutamide, harmol hydrochloride did not show any agonistic activity towards the pregnane X receptor (PXR), a master regulator of drug metabolism. Together, our results shed light on the importance of the cellular context for the screening of new AR antagonists. They further indicate that some of the potential hits that were previously identified may have been overlooked.

## 2. Materials and Methods

### 2.1. Cell Culture

Human cancer cell lines were obtained from the American Type Culture Collection (ATCC). U2OS, DU145, and VCaP cells were cultured in Dulbecco’s Modified Eagle (DMEM) medium, and PC3 and LNCaP-FGC cells were cultured in RPMI-1640 medium supplemented with 10% fetal calf serum without antibiotics. HG5LN (HeLa GAL4REx5-luciferase) and HELN (HeLa ERE-luciferase) cells were cultured in DMEM/F-12 medium supplemented with 5% fetal bovine serum, 100 units/mL of penicillin, 100 µg/mL of streptomycin and 1 mg/mL geneticin. HELN and HGLN5 cells stably expressing MR, PR, GR, and PXR were cultured in the same medium, supplemented with 0.5 µg/mL puromycin. All cell lines were cultured at 37 °C under 5% CO_2_.

High throughput screens and antagonism tests were performed in DMEM, supplemented with 5% charcoal-stripped serum medium in the presence of 1% penicillin and streptomycin.

### 2.2. Compound Library and Reagents

High-content screening was performed using the Prestwick Phytochemical Library (Prestwick Chemical, Illkirch, France), a collection of 320 pure natural products, mostly derived from plants. Harmol hydrochloride was obtained from Santa Cruz Biotechnology, Inc. Harmine, harmane, R1881, spironolactone, RU486, R5020, dexamethasone, and aldosterone were purchased from Sigma Aldrich (St. Quentin Fallavier, France). Enzalutamide was purchased from Selleckchem (Euromedex, Strasbourg, France), SR12813 from Bio-Techne (Lille, France). All reagents were dissolved in dimethyl sulfoxide (DMSO) at 10 mM before use.

### 2.3. Stable Cell Lines

**Figure 1:**
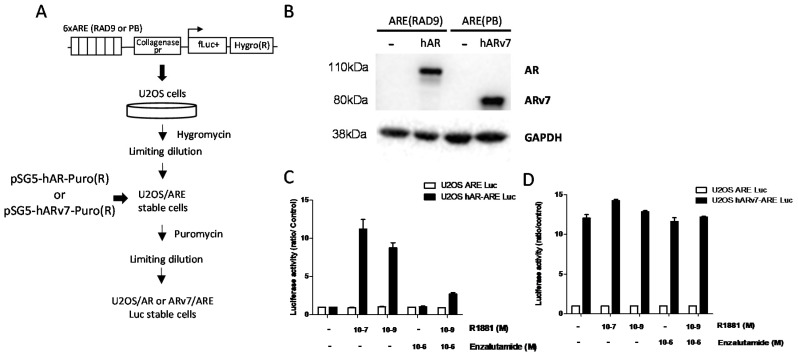
The U2OS osteosarcoma cell models used for this study. (**A**) Workflow of the generation of U2OS cells stably expressing full-length AR or ARv7. Cells were transfected with the firefly luciferase (fLuc+) reporter plasmid, under the control of androgen responsive element carrying a hygromycin resistance marker, and stable clones were selected by limiting dilutions. Then, cells were transfected with a plasmid encoding the androgen receptor (AR), or ARv7 carrying a puromycin resistance marker, and stable clones were selected by limiting dilutions. (**B**) Representative immunoblots (*n* = 3) of full-length AR or ARv7 in U2OS-hAR-ARE-Luc, U2OS-hARv7-ARE-Luc, and U2OS-ARE-Luc control cells. GAPDH was used as a loading control. (**C**,**D**) Modulation of full-length AR and ARv7 transcriptional activity by R1881 and enzalutamide, as evaluated by luciferase activity. Results of 3 independent experiments (± SEM) are expressed as fold change as compared to controls set at 1.

Osteosarcoma U2OS reporter cell lines were generated by a two-step transfection procedure. First, cells were electroporated with a plasmid encoding the firefly luciferase reporter gene, under the control of 6 copies of the ARE(Rad9) (CCAAGGCTCTGGTAGTTCTTGGA) or ARE(PB) (AATAGGTTCTTGGAGTACTTTAC) inserted in the collagenase-luciferase-hygromycine plasmid (Figure 1A). U2OS cells were then treated with hygromycin (0.25 mg/mL) and ARE-Luc clones were isolated by limiting dilutions. Stable clones were subsequently transfected with the pSG5-hAR-Puro(R) plasmid encoding full length wild-type AR or with the pSG5-hARv7-Puro(R) plasmid encoding its ARv7 spliced variant (Figure 1A). Cells were then treated with puromycin (0.5 µg/mL) and hAR and hARv7 ARE-Luc clones were selected by limiting dilutions. These stable clones were maintained in the presence of hygromycin/puromycin, and checked for AR or ARv7 expression and their level of transcriptional activity. PC3 hAR MMTV-luc cells, also known as PALM cells, stably overexpressing the full length AR were obtained as previously described, and are maintained in the presence of 0.5 µg/mL puromycin and 1 mg/mL G418 [32].

HG5LN MR and HG5LN PXR luciferase reporter cell lines were obtained by stable expression of individual ligand binding domains fused to GAL4 DNA binding domain in HG5LN (HeLa GAL4REx5-luciferase) cells, as previously described [28,33,34]. HELN PR cells were obtained by stably expressing PR with the ERα DNA binding domain in HELN (HeLa ERE-luciferase) cells and HMLN GR cells were obtained by stable co-transfection of HeLa cells with a plasmid encoding for a glucocorticoid responsive gene (MMTV-Luc-SV-Neo) and a glucocorticoid receptor expressing plasmid, as previously described [28].

### 2.4. Transactivation Assays

U2OS reporter cells stably expressing hAR (U2OS-hAR-ARE-Luc), or hARv7 (U2OS-hARv7-ARE-Luc), and U2OS-ARE-Luc control cells were plated in clear-bottomed 96-well plates in DMEM, supplemented with 10% FBS at 80% of confluence. The day after, the medium was replaced by DMEM without phenol red, supplemented with 5% charcoal-stripped serum in the presence of 100 units/mL of penicillin and 100 μg/mL of streptomycin. Each compound from the library was then added to U2OS-hAR-ARE-Luc and U2OS-ARE-Luc cells at 4 concentrations (0.3, 1, 3, and 10 µM) for an additional 16 h at 37 °C, in the presence of 1 nM R1881 that corresponds to a suboptimal concentration, inducing 80% agonistic activity. We used R1881 because it is less subject to metabolism compared to dihydroxytestosterone (DHT). The medium was then replaced with a test medium containing 0.3 mM luciferin, and luminescence was measured using a MicroBeta Wallac luminometer (PerkinElmer, Waltham, MA, USA). Screening were performed in duplicate in two separate experiments, and data were expressed as % of the maximal activity obtained with 100 nM R1881 alone. Enzalutamide (1 µM) was used as a positive control. The antagonistic activity of the positive hits towards hAR was validated using the same protocol in the absence of R1881, or in the presence of 1 or 100 nM of the agonist, corresponding to approximately 80% and maximal luciferase activity, respectively. Tests were performed in triplicates using 6 concentrations (0.01 to 3 µM) of each compound, and the results were expressed as percentages ± SEM of the luciferase activity obtained in the presence of 10 nM R1881. 

Screening for ARv7 inhibitors was performed using U2OS-hARv7-ARE-Luc and U2OS-ARE-Luc control cells with the same protocol in the absence of R1881.

Transactivation assays to evaluate the activities of harmol and enzalutamide on other nuclear receptors were performed using the same protocol as for U2OS cells. Agonistic activities were evaluated in HMLN GR, HELN PR, HG5LN MR, and HG5LN PXR cells, using dexamethasone 100 nM, R5020 100 nM, aldosterone 100 nM, and SR12813 3 μM as a positive control, respectively. Antagonistic activity towards GR, PR, MR, and PXR were evaluated by co-incubating increasing concentrations of the tested compound with a concentration of agonist that induced 80% of maximal luciferase activity, i.e., 1 nM R5020 (for PR), 5 nM dexamethasone (for GR), 1 nM aldosterone (for MR), 300 nM SR12813 (for PXR). RU486 was used as a control antagonist for GR and PR, and spironolactone for MR.

### 2.5. Cytotoxicity Assays in Spheroid Cultures

Spheroids were generated by seeding prostate cancer cells in non-adherent U-bottom 96-well plates in medium supplemented with 5% of charcoal stripped FBS. VCaP, LNCaP-FGC, DU145, or PC3 cells were seeded in duplicate at a density of 2000, 500, 600, and 600 cells per well, respectively. Three days after, spheroids were treated with indicated concentrations of the drugs in the presence of 1 nM R1881, and both cell death and proliferation were assessed 7 days later. Cell death was first evaluated by the incubation of the spheroids with propidium iodide (1 mg/mL) for 30 min, and quantitation of red fluorescence was performed using a Celigo^®^ imaging cytometer (Nexcelom Bioscience). The results (*n* = 3 ± SEM) were expressed as mortality ratios (number of dead cells in treated samples vs. untreated controls) as a function of drug concentrations. Then, proliferation was assessed following cell lysis with the CellTiter-Glo^®^ Luminescent Cell Viability Assay reagent (Promega, Charbonnière, France), according to the manufacturer protocol. Quantification of the luminescence was performed using a MicroBeta Wallac luminometer (PerkinElmer), and percent growth was calculated relative to DMSO treated cells. IC_50_ values were determined using the Prism^®^ software, and are the results of 3 independent experiments.

### 2.6. RT-qPCR 

LNCaP and VCaP cells were seeded in 6-well plates (10^6^ and 2 × 10^6^ cells per well, respectively) and, 48 h later, complete medium was replaced by charcoal-stripped medium with 5% FBS. Cells were then treated for 24 h with the tested compounds, and RNA extraction was performed using the Quick RNA Miniprep kit (Zymo-Research, Irvine, CA, USA), according to the manufacturer’s protocol. Reverse transcription was performed with 0.5 µg total RNA using the SuperScript III reverse transcriptase kit (ThermoFisher, Waltham, MA, USA), according to the manufacturer’s protocol, in a final volume of 20 µL. The levels of genes transcripts were measured by real time PCR using a LightCycler 480 system (Roche Life Science, Penzberg, Germany) in the presence of SYBRGreen (SYBR^®^ Premix Ex Taq^tm^) and the following primers: FKBP5, sense 5′-GCGGCGACAGGTTCTCTACTT-3′; antisense 5′-TCATCGGCGTTTCCTCACCA-3′; PSA, sense 5′-CCCTGTCCGTGACGTGGATT-3′; antisense 5′-CAGCAAGATCACGCTTTTGTTCC-3′; TMPRSS2, sense 5′-GGACAGTGTGCACCTCAAAGAC-3′, antisense 5′-TCCCACGAGGAAGGTCCC-3′; GR, sense 5′-ACAGCATCCCTTTCTCAACAG-3′, antisense 5′-AGATCCTTGGCACCTATTCCAAT-3′; HPRT, sense 5′-CTGACCTGCTGGATTACA-3′; antisense 5′-GCGACCTTGACCATCTTT-3′; GAPDH, sense 5′-AATTGAGCCCGCAGCCTCCC-3′; antisense 5′-CCAGGCGCCCAATACGACCA-3′. Expression levels were evaluated with the ΔΔCt method and were normalized to GAPDH. Results are expressed as mRNA expression, relative to control (untreated cells), and are the mean ± SEM of 3 independent experiments.

### 2.7. Western Blotting

U2OS cells were trypsinized and cell pellets lysed in 1 volume of 1X lysis buffer (10 mM Tris pH 7.4, 150 mM NaCl, 1 mM EDTA, 1% Triton X100, 0.5% NP-40) for 30 min at 4 °C and centrifuged for 15 min at 15,000 g and 20 µg of proteins from the supernatants were electrophoresed on a 10% SDS-PAGE gels and transferred onto nitrocellulose membranes. Immunoblotting was performed using the following antibodies: AR (D6F11 from CST, 1/1000), GR (D6H2L from CST, 1/1000), and GAPDH (14C10 from CST, 1/5000), and proteins were visualized by chemiluminescence detection using the ECL RevelBlot Plus or RevelBlot Intense (Ozyme, Paris, France).

### 2.8. Whole-Cell AR Competitive Binding Assays

U2OS hAR-AREluc cells were seeded at a density of 80,000 cells per well in clear-bottomed 96-well plates (Greiner Bio-One, Courtaboeuf, France). The day after, cells were labeled with 0.3 nM [^3^H]-R1881 (41.3 Ci/mmol specific activity) in the absence or presence of enzalutamide, harmol (0.01–10 μM), or unlabeled R1881 (0.01–300 nM) for 3 h at 37 °C, and washed three times with 200 μL of cold PBS, to eliminate unbound material. Cells were then lysed with 50 μL of LSC-cocktail (Emulsifier-Safe, Packard BioScience, Wellesley, MA, USA), and [^3^H]-bound radioactivity was counted in scintillation liquid with a microbeta trilux (Perkin Elmer, Wellesley, MA, USA). Non-specific binding was determined in the presence of 300 nM unlabeled R1881. Specific binding was calculated by subtracting non-specific binding from the total binding, and expressed as disintegrations per minute (dpm). The results were plotted as % of maximal [^3^H]-R1881 specific binding, which was obtained in the absence of a competitor (250–300 dpm) and set at 100%. IC_50_ values were defined as the concentrations required to decrease maximal [^3^H]-R1881 binding by 50%. Relative binding affinities (RBA) were calculated as IC_50_ ratios between each competitor and R1881. The RBA value for R1881 was arbitrarily set at 100.

## 3. Results

### 3.1. High-Throughput Screening of AR and ARv7 Inhibitors

In order to identify compounds that could impair the transcriptional activity of AR or of its splice variant ARv7, we used the human U2OS osteosarcoma cells that were stably transfected with each form of the receptor, and a plasmid encoding the luciferase reporter gene under the control of AR responsive elements (ARE) (Figure 1A). 

Besides the fact that they can be easily transfected, U2OS cells were chosen because they are genetically stable, and because the basal levels of expression of AR and other nuclear receptors are very low [27]. We verified that cells transiently transfected with hAR could respond to AR transactivation by the synthetic androgen R1881, using two reporter plasmids with two different ARE, Rad9 or Probasin (PB). R1881 could enhance the transcriptional activity of hAR by ~10-fold, regardless of the reporter plasmid, with a slightly better effect for the ARE RAD9 sequence (Supplementary Figure S1), whereas transcriptional activity of ARv7 was constitutive and resulted in much higher luciferase activity (16-fold vs. 4-fold) when ARE PB sequence was used (Supplementary Figure S1). Stable U2OS-hAR-ARE(Rad9)-Luc, or U2OS-hARv7-ARE(PB)-Luc cells were then selected accordingly, along with their U2OS-ARE-Luc controls, and were validated for both AR and ARv7 expression at the protein level (Figure 1B), and at the functional level for the transcriptional activity of their receptors (Figure 1C,D). As expected, the transcriptional activity of AR was stimulated by R1881 in a dose-dependent manner, and was inhibited by the AR antagonist enzalutamide, while both R1881 and enzalutamide had no effect on ARv7 transcriptional activity. 

**Figure 2:**
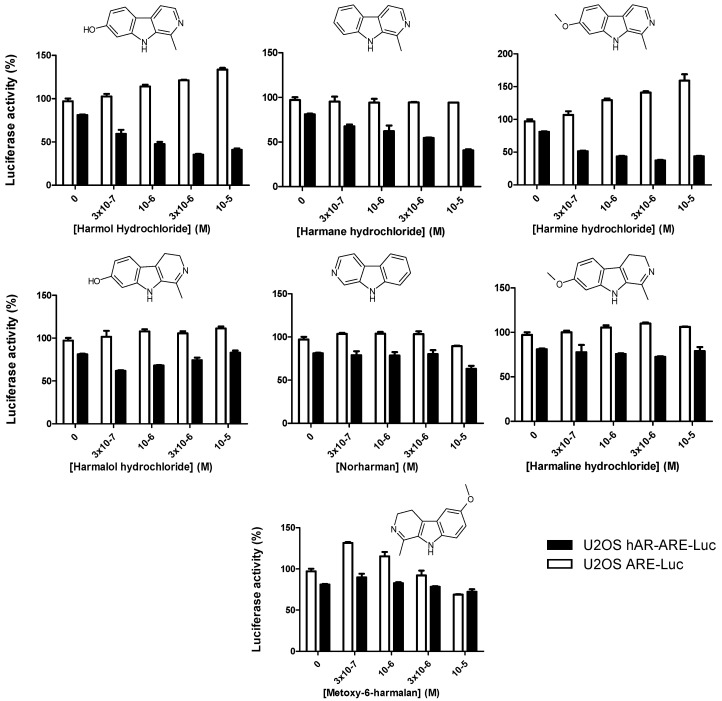
Results of the high throughput screening for harmol hydrochloride and its beta-carboline derivatives. The high throughput assays were performed in duplicate as described in the Materials and Methods section in the presence of 1 nM R1881. The effects of harmol, harmane, harmine, harmalol, norharman, harmaline, and metoxy-6-harmalan used at 0.3, 1, 3, and 10 µM on the luciferase activity (% as compared to cells treated with 100 nM R1881) in U2OS-hAR-ARE-Luc (black bars) and U2OS-androgen response elements (ARE)-Luc control cells (white bars) are shown. The chemical structures of each compound are presented above each graph.

U2OS-hAR-ARE(Rad9)-Luc and U2OS-hARv7-ARE(PB)-Luc were then used to screen the Prestwick phytochemical library^®^, containing 320 natural compounds, at four concentrations (0.3, 1, 3, 10 µM) in duplicate. The effects on AR or ARv7 transcriptional activity were measured as percentages of luminescence, compared to cells treated with 0.1 µM R1881 alone or untreated cells, respectively. Positive hits were considered when tested compounds could reduce AR- or ARv7-mediated transcriptional activity by more than 50% in a dose-dependent manner, and had no or minor effect (<20%) in control cells. Compounds for which luciferase activity was impaired in U2OS-ARE-Luc control cells were excluded. Using these criteria, we could identify nine potential hits as active inhibitors of AR transcriptional activity (2.8%): remerine, honokiol, digitoxigenin, strophantin, monensin, ellipticine, harmol, harmane, and harmine (Supplementary Table S1). Interestingly, the latter three compounds are alkaloids from the beta-carboline family, and are structurally similar to other members of this family that were found inactive: harmaline, harmalol, norharman, and methoxy-6-harmalan (Figure 2). 

Our screen also identified 10 potential activators of AR that were not studied further (Supplementary Table S1).

The same type of screening was also performed in U2OS-hARv7-ARE-Luc, but none of the 320 compounds could significantly affect the transcriptional activity of ARv7.

### 3.2. Harmol Hydrochloride is a Competitive Antagonist of Full-Length AR

**Figure 3:**
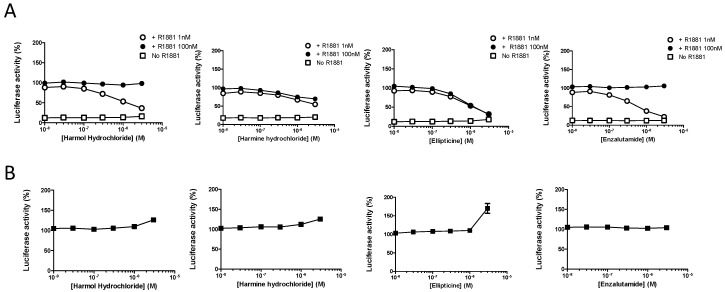
Harmol is a competitive antagonist of the full-length AR. Dose response curves performed with harmol, harmine, ellipticine and the AR antagonist enzalutamide antagonist activity in U2OS-hAR-ARE-Luc cells (**A**) and U2OS-ARE-Luc control cells (**B**). Cells were incubated for 16 h with 0, 0.03, 0.1, 0.3, 1, and 3 µM of tested compounds in the absence (□) or in the presence of R1881 1 nM (○) and100 nM (●) R1881.

Validation of the potential inhibitors of AR was then performed by testing the different hits at six concentrations in the absence or in the presence of 1 nM, or an excess (100 nM) of the synthetic agonist R1881 using the same protocol. Only harmol, harmine, and ellipticine could impair the transcriptional activity of AR (Figure 3A), while they had no effect on the luciferase activity in U2OS-ARE-Luc control cells (Figure 3B).

In order to assess the competitive nature of these inhibitors, we used the known AR competitive antagonist enzalutamide as a positive control. As shown in Figure 3A, enzalutamide inhibited AR transcriptional activity in the presence of 1 nM R1881 in a dose-dependent manner, and did not prevent the maximal effect that was observed for 100 nM R1881. A similar pattern of AR inhibition was observed for harmol hydrochloride, indicating a competitive antagonism, a result that is not in accordance with what was reported previously [25] (Figure 3A). Harmine hydrochloride and ellipticine could also exert an antagonistic activity. However, this activity was similar whether R1881 was used at 1 nM or 100 nM R1881, suggesting that, conversely to harmol hydrochloride, these compounds inhibited AR in a non-competitive manner (Figure 3A).

In order to confirm the competitive nature of harmol antagonism in a prostate cancer context, we tested the antagonist effects of harmol in PC-3 cells stably overexpressing AR (PALM cells also referred to as PC3 hAR MMTV-luc cells), in the presence of various concentrations of R1881 (0.1, 0.3, 1, 10 nM) using the same transactivation assays (Supplementary Figure S2). As was observed in U2OS cells, harmol could also inhibit AR transactivation in PC3 hAR MMTV-luc cells with the following IC_50_s: 183 nM (R1881 0.1 nM), 535 nM (R1881 0.3 nM), and 1.9 µM (R1881 1 nM). As anticipated, inhibition was not significant in the presence of 10 nM R1881. Similar results were obtained in DU145 cells transiently overexpressing the full-length AR, with a significant inhibition of AR transactivation in the presence of 1 nM R1881 (Supplementary Figure S2). Together, these results confirm that harmol hydrochloride is a competitive inhibitor of AR.

**Figure 4:**
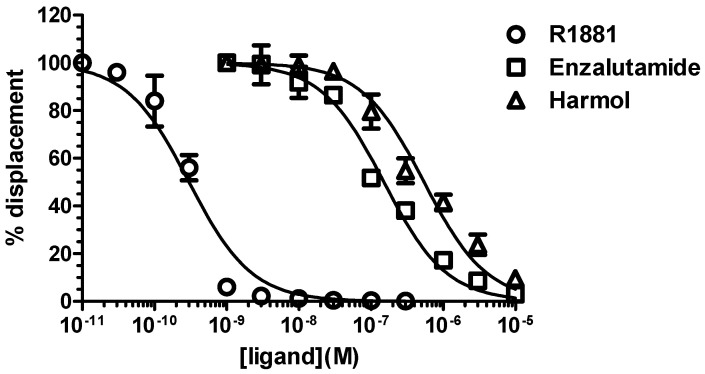
Harmol hydrochloride binds to AR. U2OS-hAR-ARE-Luc cells were incubated with 0.3 nM [^3^H]-labeled R1881 at 37 °C for 3 h in the absence or presence of enzalutamide, harmol hydrochloride (0.01–10 µM), or unlabeled R1881 (0.01–300 nM). Non-specific binding was determined in the presence of 300 nM unlabeled R1881. Specific binding was calculated by subtracting non-specific binding from total binding. Results were plotted as % of maximal [^3^H]-labeled R1881 binding as a function of drug concentrations (*n* = 3 ± SEM).

To further strengthen the fact that harmol hydrochloride is a competitive antagonist of AR, we studied its potential binding to AR in U2OS-hAR-ARE-Luc cells that were incubated with 0.3 nM [^3^H]-R1881, and increasing concentrations of unlabeled R1881 or of the tested drugs (Figure 4).

The results showed that both unlabeled R1881 and enzalutamide could compete with labeled R1881 for AR binding. They also demonstrated that harmol hydrochloride could compete with the ligand for binding with an IC_50_ of 540 nM vs. 150 nM for enzalutamide, demonstrating its affinity for the receptor (Figure 4).

### 3.3. Harmol Hydrochloride Inhibits the Growth of AR Positive Prostate Cancer Cells

**Figure 5:**
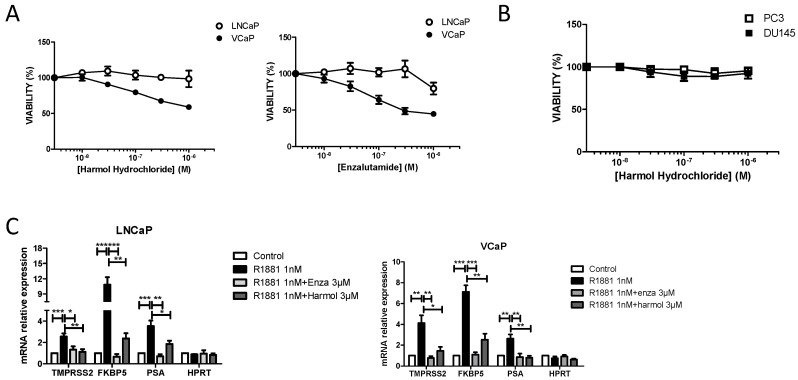
Harmol-mediated growth inhibition of LNCaP and VCaP cells is associated with the repression of AR target genes. (**A**) Effects of harmol hydrochloride and enzalutamide on the growth of LNCaP and VCaP spheroids. (**B**) Effects of harmol hydrochloride on the growth of PC3 and DU145 spheroids. Spheroids were treated for 7 days, and cell death was evaluated by propidium iodide staining and fluorescence quantification, using the Celigo imaging system. Growth inhibition was then assessed by cell lysis with CellTiter-Glo, and quantification of the luminescence using a MicroBeta Wallac luminometer. Percent growth was calculated relative to dimethyl sulfoxide (DMSO) treated cells (*n* = 3 ± SEM). (**C**) Effects of harmol hydrochloride and enzalutamide on the expression of AR target genes TMPRSS2, FKBP5, and PSA in LNCaP and VCaP cells. Cells were treated for 24 h with 3 µM harmol hydrochloride or enzalutamide in the presence of 1 nM R1881, and gene expression was evaluated by RT-qPCR. The HPRT gene was used as an AR non-target gene. Results are expressed as mRNA relative expression, as compared to untreated spheroids (*n* = 4 ± SEM). * *p* ≤ 0.05; ** *p* ≤ 0.01; *** *p* ≤ 0.001.

We then compared the antiproliferative activity of harmol hydrochloride with enzalutamide, using spheroids from VCaP or LNCaP prostate cancer cell lines. LNCaP cells endogenously express a mutated form of AR (T877A) in its LBD, which is known to affect steroid binding characteristics and response to antiandrogens. VCaP cells express both the wild-type form of AR and its ARv7 variant, though the wild-type form is predominantly expressed at the basal level (Supplementary Figure S3). The results show that enzalutamide could inhibit the growth of VCaP and LNCaP cells, tough to a lesser extent, when concentrations of enzalutamide compatible with AR antagonism (0.1 to 1 µM) were used (Figure 5A). These results are in accordance with previous studies using 2D cultures [35,36,37,38,39].

A similar effect was observed for harmol hydrochloride, though higher concentrations of the drug were required, which is in accordance with its lower affinity for AR. Conversely, harmol hydrochloride had no effect on the growth of DU145 or PC3 cancer cell lines that do not express AR (Figure 5B). We further showed that growth inhibition of LNCaP and VCaP spheroids was due to a cytostatic effect of the drugs as both enzalutamide and harmol hydrochloride did not significantly affect the number of propidium iodide positive cells as compared to untreated spheroids, even for the highest concentrations used (Supplementary Figure S3).

As it was already demonstrated for enzalutamide, we found that growth inhibition of VCaP and LNCaP spheroids induced by harmol hydrochloride was accompanied by a significant decrease of R1881-mediated expression of well-known AR target genes, such as TMPRSS2, FKBP5, and PSA, whereas the expression of the AR non-target gene HPRT was unaffected (Figure 5C). In accordance with binding experiments, the decrease in AR-target gene expression was more pronounced for enzalutamide than for harmol hydrochloride (Figure 5C).

### 3.4. Harmol Hydrochloride Is a “Selective” Antagonist of the Androgen Receptor

**Figure 6:**
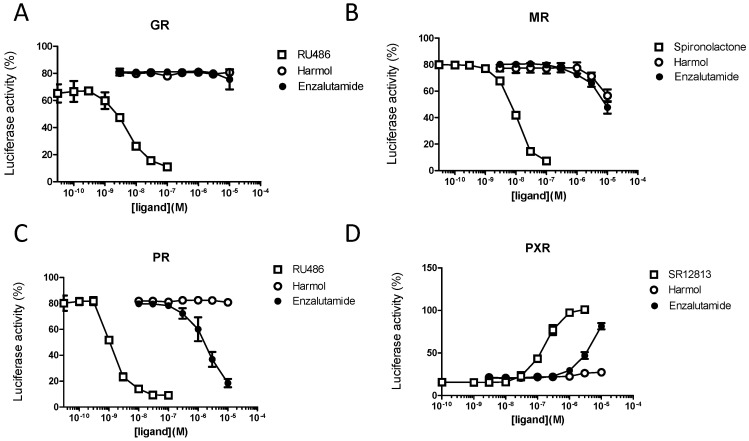
Harmol hydrochloride is a selective AR antagonist and does not activate the pregnane X receptor (PXR). HMLN glucocorticoid receptor (GR), HG5LN mineralocorticoid receptor (MR), HELN progesterone receptor (PR) and HG5LN PXR cells were used to evaluate the antagonistic effect of the tested compounds on (**A**) the glucocorticoid receptor (GR), (**B**) the mineralocorticoid receptor (MR), (**C**) the progesterone receptor (PR) and (**D**) the agonistic activity towards the pregnane X receptor (PXR). Each cell line was incubated for 16 h, with increasing concentrations of the indicated compound in the presence of 5 nM dexamethasone (for GR), 1 nM aldosterone (for MR) and 1 nM R5020 (for PR). RU486 and spironolactone were used as competitive antagonist controls for GR, PR, and MR, and SR12813 as an agonist control of PXR.

We then tested the effect of harmol hydrochloride and enzalutamide for their potential activities towards the progesterone (PR), glucocorticoid (GR), mineralocorticoid receptors (MR) and PXR (NR1I2), using dedicated luciferase reporter cell lines [28] (Figure 6 and data not shown).

Harmol hydrochloride did not show any significant activity towards GR, PR, and PXR. Both harmol hydrochloride and enzalutamide showed a small antagonistic activity towards MR at the highest concentrations used (>1 µM). In contrast, enzalutamide displayed antagonistic activity towards PR for concentrations ranging from 0.5 to 3 µM (Figure 6C), and could also activate PXR, though this effect was observed for concentrations 10-fold higher than those used for the reference agonist SR12813 (Figure 6D). We also tested whether harmol could indirectly modulate the expression of GR and participate in the growth inhibition effects in prostate cancer cells. The results showed that neither harmol nor enzalutamide treatment could significantly alter the expression of GR both at the mRNA and protein levels in LNCaP or VCaP cells (Supplementary Figure S4).

Altogether, our data validate the use of U2OS cell model for the screening of compounds that could alter the transcriptional activity of nuclear receptors, including the androgen receptor. They further identified harmol hydrochloride as a competitive and selective antagonist of the androgen receptor.

## 4. Discussion

Despite the approval of new generation AR antagonists, resistance to castration remains one of the major causes of prostate cancer treatment failures, emphasizing the need to identify more potent inhibitors of the androgen axis signaling. In the search for new derivatives, numerous studies using cell-based high content functional screens of chemical libraries from various origins have been performed. In these studies, the inhibition of AR activity was quantified using either the transcription of AR reporter genes [25,26], AR ligand-induced conformation change [26], or a reduction of AR nuclear localization [23] as readouts. However, the use of different reporter assays has been the subject of quite a number of caveats, including the absence of selectivity due to the expression of other nuclear receptors, such as GR, PR, or MR, which could alter the transcriptional response in an AR-independent manner [32,40,41,42,43]. Indeed, prostate carcinoma cells (LNCaP, DU145, PC3, LAPC4, C4-2) or other mammalian cancer cells (CHO, HEpG2, HEK293, CV-1, or MDA-MB-453 cells) that have been used for those screening campaigns strongly differ from their genetic backgrounds, in particular AR status, basal expression of other nuclear receptors, as well as AR cofactors [22,25,26,29,44,45,46,47].

The object of our study was to emphasize the importance of the choice of the cell model to screen for inhibitors of the AR transcriptional activity. For this purpose, we considered the previously validated U2OS osteosarcoma cell model stably transfected with AR or ARv7, and a luciferase reporter system. We confirmed that this model is highly selective to low levels of androgens, and is relevant for the absence of response to other nuclear receptors ligands and a high fold induction by AR ligands, which facilitates the identification of potential inhibitors ([27] and Figure 1). We then screened the Prestwick Phytochemical Library of 320 pure natural products. While no hit could be found in the case of ARv7, we identified three small molecules that inhibited the transcriptional activity of the full length AR: two alkaloids from the beta-carboline family, harmol and harmine hydrochloride, and the pirydocarbazole derivative ellipticine, a DNA intercalator that inhibits DNA topoisomerase 2 [48]. These results validate the use of the U2OS model, as harmol and harmine hydrochloride were also identified in a former FRET-based assay of FDA-approved compounds and natural products, using both prostate cancer LAPC4 and renal cancer HEK293 cells as screening models [26]. In a subsequent study, harmol was further characterized as a non-competitive inhibitor of AR, acting via a blockage of AR binding to its binding sites, and could compete for ligand binding only for concentrations that were 30- to 100-fold more than the concentration necessary to fully inhibit AR activity [25,26]. Conversely, our data clearly suggest that harmol hydrochloride behaved as a competitive inhibitor, as it showed the same inhibitory profile as the pure antagonist enzalutamide, and could also compete with the ligand binding at a concentration that is only 3.5-fold higher than that of enzalutamide. Furthermore, the pattern of inhibition of AR transcriptional activity by harmol hydrochloride was clearly different from its analog harmine hydrochloride, which inhibited AR transcriptional activity even in the presence of 0.1 µM R1881 (Figure 3), which is in agreement with previous studies, suggesting a non-competitive inhibition of AR [25,26]. While previous studies did not necessarily exclude that harmol hydrochloride could act as an AR antagonist [26], it is possible that other indirect mechanisms may be involved, including its interaction with AR cofactors, or via a regulation of PPARγ activity, as it was reported for harmine hydrochloride [49]. Harmol and harmine hydrochloride are inverse agonists of benzodiazepine receptors that are involved in steroid synthesis, so it is also possible that their inhibition at the peripheral level could account for the inhibition of AR-mediated prostate cancer cell growth [50,51,52]. It is interesting to note that another study using chemical genomic approach to screen for AR activation inhibitors could identify celastrol, gedunin, and their derivatives as potent compounds acting via HSP90 inhibition, but also identified that harmol hydrochloride was a weak inhibitor, which was therefore not further investigated [24].

Our study also reveals, for the first time, the high selectivity of harmol hydrochloride towards AR, as it could not inhibit or activate the other nuclear receptors of the same family. Moreover, we found that harmol hydrochloride had no effect on the activity of PXR (NR1I2), a master regulator of the expression of genes involved in drug metabolism, such as CYP3A4, a well-known cytochrome implicated in the metabolism of numerous xenobiotics, including anticancer drugs [53]. This is in contrast to enzalutamide, which was found to be an agonist of PXR at concentrations that are in the range of the Cmin that are required to saturate binding to AR i.e., 10–30 µM [54]. This may be of importance for potential drug–drug interactions, as enzalutamide is primarily eliminated by hepatic metabolism by CYP3A4 and CYP2C8, and could interfere with the metabolism of other drugs [55,56]. Harmol hydrochloride would thus represent an interesting alternative for a highly selective inhibition of AR deprived from potential drug–drug interaction side effects. Nevertheless, harmol hydrochloride was shown to be rapidly metabolized when administered in mice, and could therefore not be evaluated further in vivo [25]. Since harmol is the main metabolite of harmine in humans via its O-demethylation [57], it could be interesting to test this prodrug in vivo. Alternatively, the design of new harmol derivatives that could have a prolonged half-life in vivo could be envisaged. However, conversely to enzalutamide that prevents the AF2 helix to stabilize the receptor in its active conformation, there is currently no indication of the precise mechanism by which harmol is antagonizing AR. Indeed, harmol does not contain a bulky side chain that generally engenders antagonism by generating a steric clash with the AF2 helix. Our molecular modeling analysis shows that harmol is smaller than DHT, and is therefore engaged in a reduced number of interactions with the protein (Supplementary Figure S5). This observation is in full agreement with its weaker binding affinity relative to DHT. Our model also suggests a mechanism of “passive antagonism”, by which harmol can bind to the receptor, thereby competing with the natural ligand, but would be unable to initiate the productive interactions necessary to maintain the AR in an active conformation [58].

## 5. Conclusions

Altogether, our data confirm that U2OS osteosarcoma cells represent a pertinent model for HCS-based functional screening of potential inhibitors of the transcriptional activity of the androgen receptor. Indeed, prostate cancer cells with heterogenous expression of AR and other nuclear receptors, as well as their associated cofactors that may differ drastically between each other, could eventually mask the activity of potential hits, and may not be the most pertinent models. Our results not only identified harmol as a competitive inhibitor of AR, but also show, for the first time, the selectivity of this derivative towards AR. This finding has potential implications with respect to the use of second-generation AR antagonists, such as enzalutamide. Indeed, conversely to harmol, enzalutamide can activate PXR and lead to potential drug–drug interactions that are associated with adverse events. Moreover, the fact that harmol antagonizes AR through a mechanism that differs from enzalutamide, one can hypothesize that it could exert a different biological response. The active conformation of the AF2 helix is not sterically precluded by harmol, so it might be more dependent on the expression level of coregulators, thereby generating some cell- or tissue-specificity, and may therefore represent an interesting therapeutic alternative to currently approved AR antagonists used in the clinic.

## Supplementary Materials

The following are available online at https://www.mdpi.com/2073-4409/9/6/1469/s1, Figure S1: Choice of the AR responsive element and of the AR ligand for the screening assays, Figure S2: Harmol is a competitive antagonist of the full-length AR in PC3 and DU145 prostate cancer cells overexpressing AR, Figure S3: Harmol hydrochloride and enzalutamide are cytostatic, Figure S4: Effects of Harmol and Enzalutamide on the expression of GR, Figure S5: Modeling of harmol hydrochloride (in green) in the ligand binding pocket of AR, Table S1: Results of the screening of the Prestwick Phytochemical Library using the U2OS-hAR-ARE-Luc cell model.
