# Supplementary material for: High Content Screening Using New U2OS Reporter Cell Models Identifies Harmol Hydrochloride as a Selective and Competitive Antagonist of the Androgen Receptor
Source: Cells. 2020 Jun 16;9(6):1469. doi: 10.3390/cells9061469 (PMC7349874; doi:10.3390/cells9061469)
Supplement: Supplementary file 1 [file cells-09-01469-s001.pdf]

SUPPLEMENTARY MATERIAL

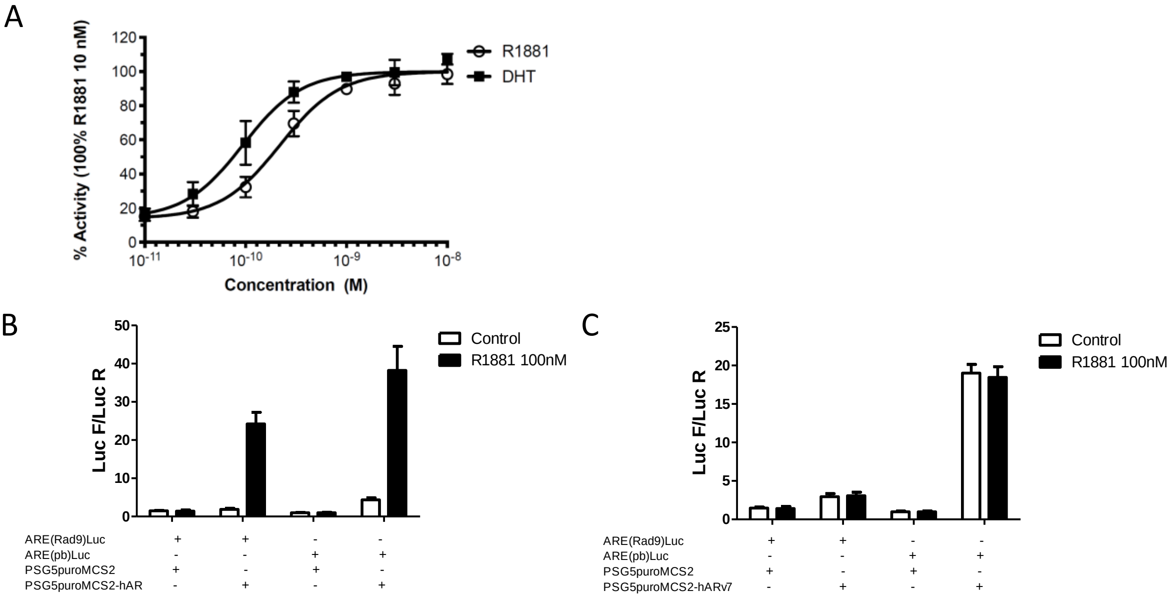

Supplementary Figure S1. **Choice of the AR responsive element and of the AR ligand for the screening assays (A)** Comparison of the agonist effect between DHT and R1881 in U2OS-hAR-ARE-Luc cells. Cells were treated with indicated concentrations of the androgens for 16h and AR transactivation was evaluated as described in Materials and Methods. **(B, C)** Comparison between the Rad9 and the probasin (PB) sequences of the AR responsive element (ARE) for the induction of AR transcriptional activity. U2OS cells were transiently transfected with a plasmid expressing hAR **(B)** or hARv7 **(C)**, a Firefly luciferase reporter plasmid containing either the Rad9 or the PB ARE and a plasmid expressing the Renilla luciferase. The next day cells were incubated for 16 h in the presence of 100 nM R1881 and the luminescence was quantified. The results were expressed as ratios between the activity of the Firefly luciferase and the Renilla luciferase.

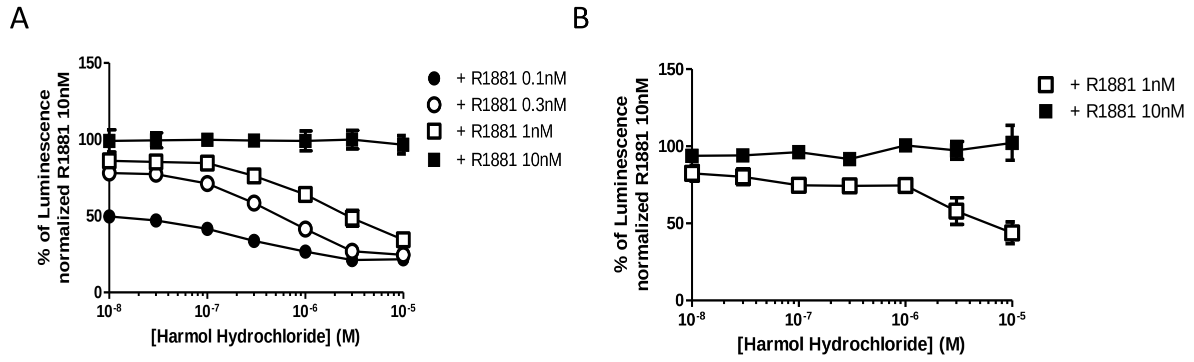

Supplementary Figure S2. Harmol is a competitive antagonist of the full-length AR in PC3 and DU145 prostate cancer cells overexpressing AR. (A) Dose response curves performed with harmol in PC3 cells stably overexpressing AR (PC3 hAR MMTV-luc) in the presence of increasing concentrations of R1881 (0.1, 0.3, 1, 10 nM) using the same transactivation assays as in Fig. 3. Results are the mean ± SEM of two independent experiments performed in quadruplicate. **(B)** Same as in **(A)** using DU145 cells (5,000 cells/100 mm^2 dish) transiently co-transfected with 10 µg of pSG5-hAR-Puro(R), 10 µg of 6xARE-PB-)-luc+-Hygro(R), and 5 µg of the pRL-CMV Renilla plasmids using lipofectamine 2000. One day later, transactivation assays were conducted as described in Materials and Methods. Results are the mean of two independent experiments performed in quadruplicate and are expressed as ratios between the activity of the Firefly luciferase and the Renilla luciferase and normalized as % compared to cells treated with R1881 at 10nM

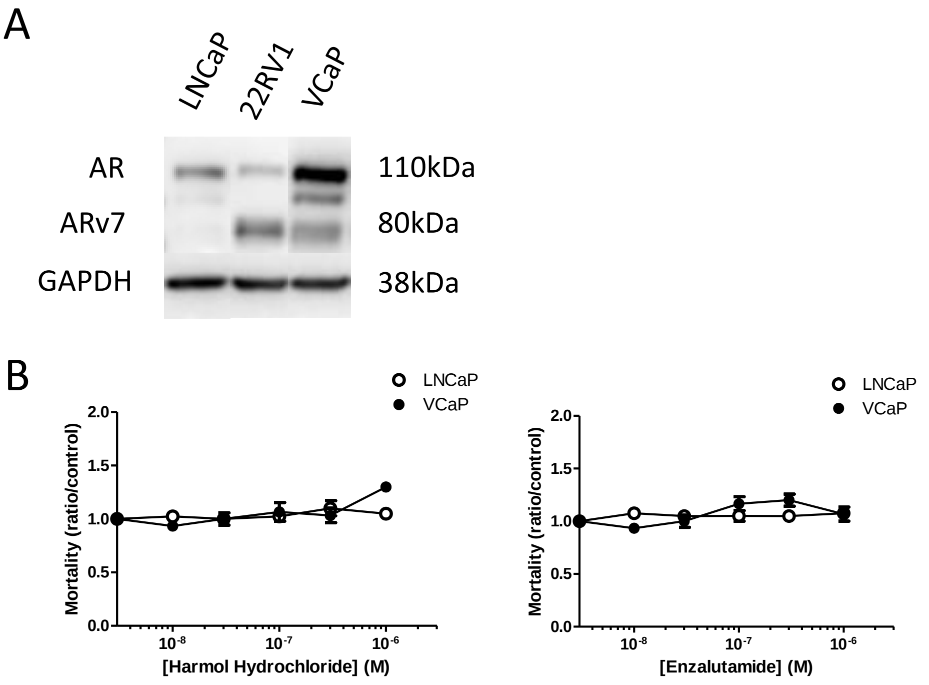

Supplementary Figure S3. Harmol hydrochloride and enzalutamide are cytostatic. (A) Western blots showing the basal expression of AR and ARv7 in LNCaP, VCaP and 22RV1 prostate cancer cells. GAPDH was used as a loading control. **(B)** Effects of Harmol hydrochloride and enzalutamide on cell death. Spheroids were treated for 7 days with the indicated concentrations of each drug, stained with PI (1 mg/mL) for 30 min and luminescence was evaluated using the Celigo® imaging system. Results are expressed as mortality ratios as compared to untreated spheroids (set at 1). Each point represents the mean ± SEM of 3 independent experiments.

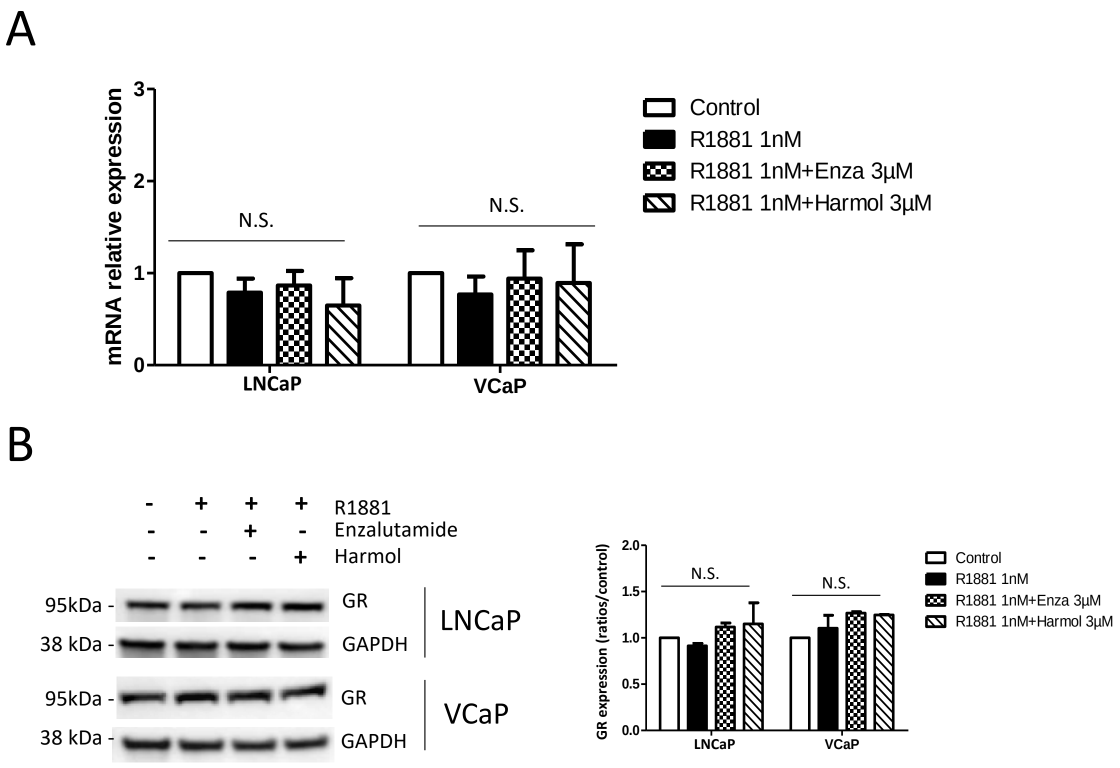

Supplementary Figure S4. Effects of Harmol and Enzalutamide on the expression of GR. LNCaP and VCaP cells were treated for 24h with 3 µM harmol hydrochloride or enzalutamide in the presence of 1 nM R1881 and GR expression was evaluated by RT-qPCR (n=3) **(A)** and by Western blotting (n=2) **(B)**. Results are expressed as means ± SEM of mRNA or protein levels relative to untreated cells; N.S. Not significant.

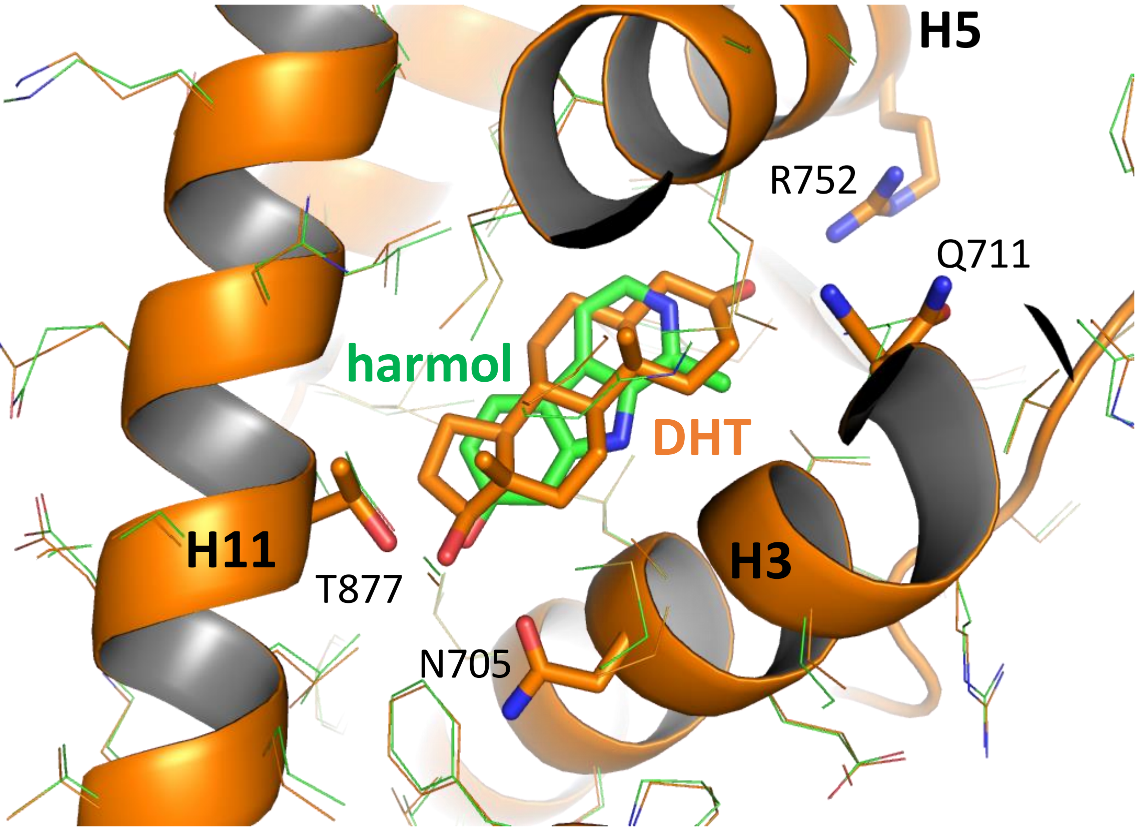

pKd 5.6

Supplementary Figure S5. Modeling of harmol hydrochloride (in green) in the ligand binding pocket of AR. For comparison, the model is superimposed on the crystal structure (PDB code 1T7R) of hAR bound to DHT (in orange). Some residues important for DHT binding are shown in stick representation and labeled. The predicted affinity of the interaction between harmol and hAR (pKd = 5.6) was obtained using the server EDMon (http://edmon.cbs.cnrs.fr).

Supplementary Table S1. Results of the screening of the Prestwick Phytochemical Library using the U2OS-hAR-ARE-Luc cell model. Positive hits were considered when tested compounds could reduce (or activate) AR-mediated transcriptional activity by more than 50% in a dose-dependent manner and had no or minor effect (<20%) in U2OS-ARE-Luc control cells.

Prestwick NAT number	Compound	Results of 1st screen on AR (Results of confirmatory screen)
0001	Aristolochic Acid	No effect
0002	Astilbin	No effect
0003	Cardamonin	No effect
0004	Cimifugin	No effect
0005	Ellagic Acid	No effect
0006	Evodiamine	No effect
0007	Genistin	No effect
0008	Hesperidin	No effect
0009	Huperzine A	No effect
0010	Indirubin	No effect
0011	Jervine	No effect
0012	Matrine	No effect
0013	Rutaecarpine	No effect
0014	Rutin	No effect
0015	Salicin	No effect
0016	Strychnine	No effect
0017	Acacetin	No effect
0018	Berlambine	No effect
0019	Betulinic acid	No effect

0022	Eleagnine hydrochloride (R,S)	No effect
0023	Betonicine	No effect
0024	Myosmine	No effect
0025	Methoxy-6-harmalan	No effect
0026	Norharman	No effect
0027	Piperlongumine	No effect
0028	Rotenone	No effect
0029	Apigenin	No effect
0030	Anisomycin	No effect
0031	Genistein	No effect
0032	Pyridostigmine bromide	No effect
0033	Kaempferol	No effect
0034	Angelicin	No effect
0035	Baicalin	No effect
0036	Cynarin	No effect
0037	Glycitin	No effect
0038	Oxymatrine	No effect
0039	Trigonelline hydrochloride	No effect
0040	2,6-Pyridinedicarboxylic Acid	No effect
0043	Evoxine	No effect
0046	Lycorine HCl	No effect
0048	Pinocembrine	No effect
0049	Remerine HCl	Inhibition (Not confirmed)
0050	Stevioside	No effect
0051	Unsevine	No effect
0052	Lapachol	No effect

0053	1,8-Dihydroxy-3-methylanthraquinone	No effect
0055	4,4'-(2,3-Dimethyltetramethylene)dipyrocatechol	No effect
0056	Xanthurenic Acid	No effect
0058	2-Hydroxy-[1,4]naphthoquinone	No effect
0059	Honokiol	Inhibition(Not confirmed)
0060	Isoliquiritigenin	No effect
0061	Kinetin riboside	No effect
0062	Curcumin	No effect
0063	beta-lapachone	No effect
0064	Precocene 2	No effect
0065	(+/-)-2-cis-4-trans-Abscisic acid	No effect
0066	Mahanimbine	No effect
0067	Abietic Acid	No effect
0068	Roseoflavin	No effect
0069	Cotinine (-)	No effect
0070	Ursolic acid	No effect
0071	R(-) Apomorphine hydrochloride hemihydrate	No effect
0072	Tomatine	No effect
0073	Bromocryptine mesylate	No effect
0074	Hesperetin	No effect
0075	Erythromycin	No effect
0076	Catharanthine	No effect
0077	Hydrastinine hydrochloride	No effect
0078	Camptothecine (S,+)	Activation

0079	Kawain	No effect
0080	Hyoscyamine (L)	No effect
0081	Vinpocetine	No effect
0082	Vincamine	No effect
0083	Quinidine hydrochloride monohydrate	No effect
0084	Colchicine	No effect
0085	Mimosine	No effect
0086	Etoposide	No effect
0087	Arecoline hydrobromide	No effect
0088	Tocopherol (R,S)	No effect
0089	Aspartic acid, N-acetyl (R,S)	No effect
0090	Biotin	No effect
0091	Calciferol	No effect
0092	Carcinine	No effect
0093	Chlorogenic acid	No effect
0094	Coralyne chloride hydrate	No effect
0095	Digitoxigenin	Inhibition(Not confirmed)
0096	Fusaric acid	No effect
0097	Ginkgolide A	No effect
0098	Pilocarpine nitrate	No effect
0099	Lactobionic acid	No effect
0100	Lysergol	Activation
0101	Menadione	No effect
0102	Muramic acid, N-acetyl	No effect
0103	Myricetin	No effect

0104	Naringenine	No effect
0105	Naringin hydrate	No effect
0106	Neostigmine bromide	No effect
0107	Strophantine octahydrate	Inhibition (Not confirmed)
0109	Piperine	No effect
0110	Methoxy-8-psoralen	No effect
0111	Puromycin dihydrochloride	No effect
0112	Azathymine, 6	No effect
0113	Artemisinin	No effect
0114	Quercetine dihydrate	No effect
0115	Resveratrol	No effect
0116	Glycocholic acid	No effect
0117	Methionine sulfoximine (L)	No effect
0118	Thiocolchicoside	No effect
0119	Parthenolide	No effect
0120	Mycophenolic acid	No effect
0121	Tubocurarine chloride pentahydrate (+)	No effect
0122	Dihydroergocristine mesylate	No effect
0123	Noscapine	No effect
0124	Syrosingopine	No effect
0125	Atropine sulfate monohydrate	No effect
0126	Eserine sulfate, physostigmine sulfate	No effect
0127	Aconitine	No effect
0128	Dihydroergotoxine mesylate	No effect
0129	Emetine dihydrochloride	No effect

0130	Ipratropium bromide	No effect
0131	Rauwolscine hydrochloride	No effect
0132	Corynanthine hydrochloride	No effect
0133	Palmatine chloride	No effect
0134	Trimethylcolchicinic acid	No effect
0135	Hydrastine	No effect
0136	Lobelanidine hydrochloride	No effect
0137	Papaverine hydrochloride	Activation
0138	Yohimbine hydrochloride	No effect
0139	Lobeline alpha (-) hydrochoride	No effect
0140	Berberine chloride	Activation
0141	Galanthamine hydrobromide	No effect
0142	Bicuculline (+)	No effect
0143	Yohimbinic acid monohydrate	No effect
0144	Laudanosine (R,S)	No effect
0145	Ajmalicine hydrochloride	No effect
0146	Calycanthine	No effect
0147	Convolamine hydrochloride	No effect
0148	Isocorydine (+)	No effect
0149	Seneciphylline	No effect
0150	Boldine	No effect
0151	Ajmaline	No effect
0152	Monocrotaline	No effect
0153	Hydrocotarnine hydrobromide	No effect
0154	(-)-Cinchonidine	No effect
0155	Eburnamonine (-)	No effect

0156	Cinchonine	No effect
0157	Canavanine sulfate monohydrate (L,+)	No effect
0158	Harmaline hydrochloride dihydrate	No effect
0159	Harmalol hydrochloride dihydrate	No effect
0160	Harmol hydrochloride monohydrate	Inhibition (Confirmed)
0161	Harmine hydrochloride	Inhibition (Confirmed)
0162	Sparteine (-)	No effect
0163	Harmane hydrochloride	Inhibition (Not confirmed)
0164	Stachydrine hydrochloride	No effect
0165	Pseudopelletierine hydrochloride	No effect
0166	Salsolinol hydrobromide	No effect
0167	Gramine	No effect
0168	Thiamine hydrochloride	No effect
0169	Riboflavine	No effect
0170	Hydroquinine hydrobromide hydrate	No effect
0171	Retrorsine	No effect
0172	Conessine	No effect
0173	Protoveratrine A	No effect
0174	Solanine alpha	No effect
0175	Butirosin disulfate salt	No effect
0176	Picrotoxinin	No effect
0177	Vitexin	No effect
0178	Solasodine	No effect
0179	Foliosidine	No effect
0180	Skimmianine	No effect

0181	Tetrandrine	No effect
0182	(d,l)-Tetrahydroberberine	No effect
0183	Deltaline	No effect
0184	Graveoline	No effect
0185	Hippeastrine hydrobromide	No effect
0186	Gossypol	No effect
0187	Delsoline	No effect
0188	Fluorocurarine chloride	No effect
0189	Austricine hydrate	No effect
0190	beta- Belladonnine dichloroethylate	No effect
0191	Heliotrine	No effect
0192	Nitrarine dihydrochloride	No effect
0193	Karakoline	No effect
0194	Napelline	No effect
0195	Fillalbin	No effect
0196	Strophanthidin	No effect
0197	Monensin sodium salt	Inhibition (Not confirmed)
0198	Mevalonic-D, L acid lactone	No effect
0199	Podophyllotoxin	No effect
0200	Cycloheximide	No effect
0201	Bergenin monohydrate	No effect
0202	Catechin-(+,-) hydrate	No effect
0203	Aminophylline	No effect
0204	Luteolin	No effect
0205	Theophylline monohydrate	No effect
0206	Theobromine	No effect

0207	Reserpine	No effect
0208	Arcaine sulfate	No effect
0209	Scopolamine hydrochloride	No effect
0210	Capsaicin	No effect
0211	Digoxigenin	Activation
0212	Cantharidin	No effect
0213	Chrysin	No effect
0214	Proxyphylline	No effect
0215	3-Acetylcoumarin	No effect
0216	Esculin Hydrate	No effect
0217	Caffeic acid	No effect
0218	Esculetin	No effect
0219	(±)-Nipecotic acid	No effect
0220	Scopoletin	No effect
0221	Gibberellic acid	No effect
0222	6-Hydroxytropinone	No effect
0223	3-Acetamidocoumarin	No effect
0224	Reserpinic acid hydrochloride	No effect
0225	Beta-sistosterol	No effect
0226	Guaiacol	No effect
0227	Harpagoside	No effect
0228	Asiaticoside	No effect
0229	Betulin	No effect
0230	Arbutin	No effect
0231	alpha-Santonin	No effect
0232	Tropine	No effect

0233	Condelphine	No effect
0234	Leucomisine	No effect
0235	(+,-)-Synephrine	Activation
0236	Menaquinone	No effect
0237	Quinic acid	No effect
0238	Caffeine	No effect
0245	3,3'-methylene-bis(4-hydroxycoumarin)	No effect
0247	5-methoxy-2-methylfuranochromone	No effect
0248	acide 4-hydroxy-3,5-dimethoxybenzoique	No effect
0249	7-glucosylombéliférone	No effect
0250	Quercétine-3-rutinoside	No effect
0255	Bakankosine	No effect
0258	Hordenine	No effect
0259	Genatropine chlorhydrate	No effect
0260	Narcéine	No effect
0261	Geneserine	No effect
0262	Colchicoside	No effect
0263	Cytisine nitrate hydrate	No effect
0264	Gelsemine	No effect
0265	9-Methoxyellipticine	No effect
0266	Isatine	No effect
0268	Podocarpic acid	No effect
0270	Meconic acid	No effect
0271	Hydrocupreine	No effect
0272	Securinine	No effect

0273	Ellipticine	Inhibition (Confirmed)
0274	Epicatechin-(-)	No effect
0275	Metanephrine hydrochloride DL	Activation
0276	Anisic acid	No effect
0277	Brucine	No effect
0278	Ephedrine HCl	Activation
0279	Genoscopolamine HBr	No effect
0280	Hydrocinnamic acid	No effect
0281	Verbenaline	No effect
0282	Tabersonine dichlorhydrate	No effect
0283	Haplopine	No effect
0284	Hydroquinidine HCl	No effect
0285	Nicotinic acid	No effect
0286	Tropic acid	No effect
0287	Genostrychnine base	No effect
0288	Ungerine nitrate	No effect
0289	Sanguinarine	No effect
0291	Homatropine HBr	No effect
0292	Thebaine HCl	No effect
0293	Coronaridine	No effect
0294	Amygdalin	No effect
0295	Phloridzin	No effect
0296	Melicopicine	No effect
0297	Strychnospermine	No effect
0298	Vindolinine dichlorhydrate	No effect
0299	Vochysine (4 dia/ énantio)	No effect

0300	Xanthevodine	No effect
0301	Veprisine	No effect
0302	Diosgenin	No effect
0303	4,2,4'-Tribenzyloxy-6'-hydroxychalcone	No effect
0304	Evoxanthine	No effect
0305	Oxime de Yohimbinone	No effect
0306	10-Methoxy-o-demethyltsilanine	No effect
0307	Olivacine	No effect
0308	Kokusaginine	No effect
0309	Ethylvanilline	No effect
0310	Glycyrrhetinic Acid	No effect
0311	Triamcinolone acetonide	No effect
0312	2-hydroxy-3-methoxy-naphtoquinone	No effect
0314	Ergotamine tartrate	No effect
0316	(-)-Quinpirole hydrochloride	No effect
0317	Delcorine	No effect
0318	Cotarnine chloride	No effect
0319	Quinine dihydrochloride	No effect
0321	Gitoxin	Activation
0322	Cryptopine	No effect
0323	Triamcinolone alcohol	No effect
0324	Isoconessimine	No effect
0325	Kainic acid	No effect
0326	Veratrine	No effect
0327	Adenosine	No effect

0328	Nicotinamide	No effect
0329	Ergobasine	Activation
0330	Khelline	No effect
0331	Chelidonic acid	No effect
0332	L-tryptophane	No effect
0334	Desoxypeganine Hydrochloride	No effect
0335	Glycyrrhizic acid	No effect
0337	L-Proline	No effect
0338	L-Methionine	No effect
0339	L-menthol	No effect
0340	Tyramin hydrochloride	No effect
0341	Cytosine	No effect
0342	Ergosterine	No effect
0343	Trichodesmine	No effect
0344	Vulpinic acid	No effect
0345	Atranorin	No effect
0346	(1'S,3S,4a'S,5a'R,10a'S)-methyl 6-methoxy-1'-methyl-2-oxo-1',4a',5',5a',7',8',10',10a'-octahydrospiro[indoline-3,6'-pyrano[3,4-f]indolizine]-4'-carboxylate	No effect
0347	Variolaric acid	No effect
0348	(+)-Usnic acid	No effect
0349	Imperialine	No effect
0350	Berbamine dihydrochloride	No effect
0351	Cytidine	No effect
